# Mechanisms of 915 MHz Microwave Thermal Treatment on Physicochemical Properties and Microbial Communities in Sugarcane Continuous Cropping Soil

**DOI:** 10.3390/microorganisms14081831

**Published:** 2026-08-19

**Authors:** Junru Mao, Yanling Wu, Yifeng Huang, Yunyun Li, Min Mo, Yanye Fan, Xianrui Chen, Zhimin Huang

**Affiliations:** Guangxi Key Laboratory of Advanced Microwave Manufacturing Technology, State Key Laboratory of Non-Food Biomass Energy, Guangxi Academy of Sciences, Nanning 530007, China

**Keywords:** 915 MHz microwave, sugarcane continuous cropping soil, soil microstructure, physicochemical properties, microbial community, functional genes

## Abstract

Long-term sugarcane monoculture triggers severe continuous cropping obstacles accompanied by notable soil microecological degradation, including nutrient immobilization, soil acidification, salinization and microbial community imbalance. Physical soil remediation via industrial microwave irradiation represents a promising approach to alleviate soil degradation. Nevertheless, the interactive variations in soil structure, fertility and microbial communities under gradient 915 MHz industrial microwave irradiation remain poorly understood. This study aimed to clarify the correlations among physicochemical properties, microbial structure and functional genes of sugarcane continuous cropping soil under microwave thermal regulation. A continuous 915 MHz microwave device with power gradients (0, 2, 4, 6, 8 kW) and a fixed irradiation duration of 10 min was adopted. Soil samples were incubated for 0, 15 and 30 weeks for comprehensive parameter determination. The results demonstrated that appropriate microwave power exerted positive regulatory effects on soil thermal intensity, aggregate disruption and microbial succession. Soil organic matter (SOM) and pH were key factors modulating the distribution of beneficial and pathogenic microorganisms. The 4 kW treatment disintegrated compact soil aggregates, activated mineral-bound nutrients, relieved soil acidification and salinization, and upregulated genes responsible for nutrient mineralization and antifungal metabolism to sustain high abundances of partial biocontrol fungi. In contrast, high-power treatments (6 kW and 8 kW) induced substantial early-stage SOM loss, reduced soil pH and aggravated salinization in the late incubation stage, thereby inhibiting symbiotic beneficial fungi. Collectively, 4 kW was the optimal microwave parameter in this study to coordinate soil structural, nutritional and microecological balance. This study provides a theoretical basis and technical guidance for the green remediation of soil plagued by sugarcane continuous cropping obstacles.

## 1. Introduction

Long-term continuous planting of sugarcane induces specific continuous cropping obstacles in soil, manifested as persistent soil acidification, continuous salt accumulation and massive proliferation of pathogenic fungi, which ultimately reduces soil fertility and leads to sustained decline in sugarcane yield [1,2]. Soil remediation is a core agronomic measure to prevent the infection of soil-borne diseases and guarantee healthy farmland production. Against the background of ecological sustainability and green agricultural development, chemical-free physical remediation technologies have become a research hotspot for restoring degraded continuously cropped farmland [3]. Traditional chemical remediation technologies have obvious drawbacks [4]; chemical disinfection easily produces pesticide residues and brings ecological risks, which conflicts with the concept of green and sustainable agriculture [5,6]. Conventional physical remediation methods generally suffer from uneven heating, high energy consumption, limited single-batch treatment scale and insufficient improvement effect on deep soil layers [7,8]. Therefore, developing efficient, environmentally friendly and scalable green soil remediation technologies is critical to alleviate sugarcane continuous cropping obstacles and realize ecological restoration of degraded sugarcane fields.

Microwave thermal treatment is an emerging physical soil remediation technology. It generates frictional heat by stimulating soil polar molecules under alternating electromagnetic fields to break soil aggregates, release immobilized nutrients and suppress harmful microorganisms. Featuring pollution-free operation, rapid heating and convenient implementation, microwave technology has been widely applied in studies on soil disinfection and soil quality improvement [9]. During microwave irradiation, soil dielectric properties dynamically change with soil water content, bulk density and temperature, further resulting in uneven soil heating [10,11]. Soil pH, soil organic matter (SOM), electrical conductivity (EC), and available nutrients jointly shape the composition of soil microbial communities. Soil microorganisms drive core biochemical processes including organic matter decomposition and nutrient cycling, and play a decisive role in maintaining soil structural stability and microecological health [12]. However, inappropriate microwave power will cause soil organic matter loss, disturb microbial communities and destroy soil microecological balance. To date, the long-term dynamic response mechanisms of soil physicochemical properties and microbial communities to gradient microwave treatment remain unclear.

At present, soil microwave treatment equipment at home and abroad can be mainly divided into two categories: small rotary microwave ovens and self-developed fixed-frequency microwave devices, with 2450 MHz and 915 MHz as mainstream frequencies [13,14,15,16]. Small microwave ovens have small cavity volume and cumbersome manual operation, and are only suitable for simulation experiments with a small amount of soil samples in laboratories [17]. Most self-developed fixed-frequency devices require microwave probes to be inserted into soil for operation, accompanied by practical limitations such as small single-batch treatment volume and unbalanced thermal stability [18]. Both types of equipment can only carry out basic laboratory simulation and cannot simulate large-scale field remediation conditions for sugarcane fields. Li et al. [19] treated black soil in Northeast China with 2450 MHz microwaves for 3–12 min. The results showed that microwaves could significantly alter soil physicochemical properties and microbial community structure; beneficial microorganisms recovered faster in soils subjected to short-duration microwave treatment (3–6 min), which facilitated the prevention and control of soil-borne diseases and improvement of crop quality. Maynaud et al. [20] treated alluvial grassland soil with 915 MHz microwaves and adopted brome seeds as internal standards to evaluate sterilization efficiency. Treatments of 2 kW for 8 min and 4 kW for 4 min completely inhibited brome germination and substantially reduced microbial biomass; meanwhile, the contents of soil organic carbon and inorganic phosphorus increased by 1.6 times and 1.2 times, respectively. Chen et al. [21] proposed that soil microbial communities could basically restore their original dynamic balance within 30 days after microwave irradiation. Compared with 2450 MHz microwaves, 915 MHz microwaves possess longer wavelengths, and their penetration depth in soil exceeds three times that of the former. Although the heating rate is relatively moderate, heat distribution inside soil is more uniform, making them more suitable for deep thermal treatment of thick bulk materials [22]. Nevertheless, most of the above studies adopted short-term static treatment relying on laboratory equipment with short incubation periods, failing to link the results with the sugarcane growth cycle (30–40 weeks). Up to now, no research has taken sugarcane continuous cropping soil as the research object to explore the long-term dynamic variations in soil physicochemical properties and microbial communities under gradient-power continuous industrial microwave treatment, which greatly restricts the practical popularization and application of microwave green remediation technology [23].

Based on the above research background, this paper puts forward the core research hypothesis: Moderate-power 915 MHz continuous industrial microwave treatment can effectively alleviate soil acidification and salinization, activate soil nutrients, and reconstruct a healthy soil microbial community. On the basis of this hypothesis, typical continuously cropped sugarcane soil was used as test material in this study. A 915 MHz continuous industrial microwave system was adopted with a fixed treatment duration of 10 min, and five power gradients of 0, 2, 4, 6 and 8 kW were set. To simulate the actual sugarcane growth cycle, soil samples were collected at 0, 15 and 30 weeks after microwave treatment. Soil physicochemical properties and microbial community structure were comprehensively determined to systematically clarify the regulatory mechanisms of microwave power on soil structure, nutrient availability, microbial community assembly and functional metabolic pathways. This study aims to reveal the long-term dynamic response patterns of soil physicochemical indicators and microbial characteristics to gradient microwave treatment, clarify the ecological mechanism of industrial microwave remediation for sugarcane soil with continuous cropping obstacles, and screen the optimal microwave process parameters. The results can provide a theoretical basis and technical reference for the green ecological restoration and sustainable utilization of degraded sugarcane farmland.

## 2. Materials and Methods

### 2.1. Sample Collection

Soil samples were collected from the sugarcane experimental base of Guangxi Academy of Agricultural Sciences, Nanning, China (22°51′ N, 108°17′ E; elevation approximately 120 m). The region belongs to the south subtropical monsoon climate with an annual average rainfall of 1305 mm, and rainfall is mainly concentrated from May to September. The experimental field has been continuously monocultured with sugarcane since early spring 2016. The soil is classified as latosol (lateritic red soil), the primary soil type for sugarcane planting in southern Guangxi. A multi-point random sampling method was used to collect topsoil from the 0–15 cm plough layer. After sampling, visible stones, residual sugarcane roots and other debris were manually removed from the soil. All subsamples were fully mixed to prepare composite soil and then sieved through a 2 mm mesh screen. A portion of the homogenized soil was sealed and stored at −80 °C for microbial sequencing, and another portion was reserved for subsequent microwave treatment. A small amount of raw soil was retained to determine the initial physicochemical background values.

### 2.2. Experimental Procedures

**Figure 1:**
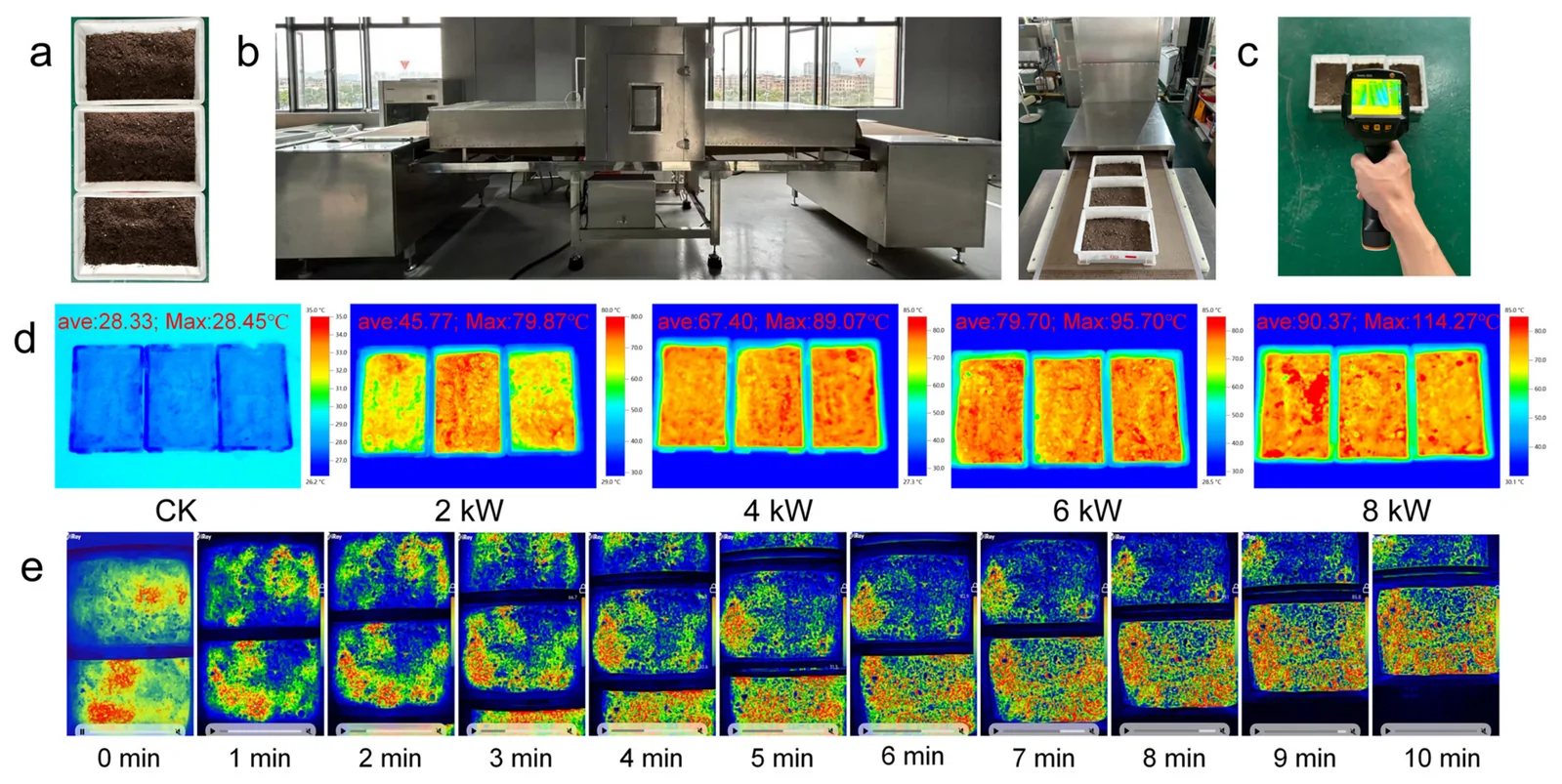
Experimental soil, equipment and temperature monitoring. (**a**) Test soil; (**b**) 915 MHz continuous microwave soil heating device; (**c**) Handheld thermal imager (Testo-883) for soil temperature measurement; (**d**) thermal maps of average and maximum soil temperatures under different microwave powers; (**e**) soil temperature changes within 0–10 min under 2 kW microwave treatment.

This experiment adopted a 915 MHz continuous industrial microwave heating device equipped with a digital power control module, real-time infrared temperature measurement system and automatic conveying belt (Figure 1b). The microwave power was continuously adjustable within 0–10 kW, which enabled stable and uniform heating of bulk soil and simulated field continuous operation conditions. Prior to treatment, impurities were removed from the air-dried soil, followed by full mixing and homogenization. Exactly 2.00 kg of prepared soil was weighed and evenly spread into a 20 × 30 cm plastic tray (Figure 1a). The initial soil temperature was measured by a Testo-883 handheld infrared thermal imager (Figure 1c, Testo-883, Testo SE & Co. KGaA, Titisee-Neustadt, Germany). The tray filled with soil was placed on the conveyor belt and sent into the microwave cavity at a constant speed for thermal treatment. According to pre-experiment results, microwave power above 10 kW or treatment time exceeding 10 min would cause severe carbonization of soil organic matter, irreversible loss of nutrients and excessive energy consumption. Therefore, the treatment duration was fixed at 10 min, and five power gradients were set: 0 kW (control group, CK), 2 kW, 4 kW, 6 kW, 8 kW. The built-in infrared sensor of the equipment recorded dynamic soil temperature data during the whole 0–10 min treatment process, and the terminal temperature of each group was measured immediately after microwave irradiation. Each treatment contained three independent biological replicates, with all soil batches treated separately to avoid cross-contamination. After microwave treatment, the soil samples were placed in a ventilated room and cooled naturally to room temperature. All cooled soil from each replicate was divided into two portions. One portion was immediately sealed in sterile plastic bags and stored in an ultra-low-temperature refrigerator at −80 °C for the extraction of total soil microbial DNA. The other portion was transferred to a constant-temperature incubator and incubated at 30 °C. Soil samples were collected at three incubation stages (0, 15 and 30 weeks). At each sampling time point, a fraction of fresh soil was preserved at −80 °C for high-throughput microbial sequencing, and the remaining collected soil was air-dried indoors, ground and sieved for the characterization of soil microstructure and the determination of soil physicochemical properties.

Regarding sample labels, the prefixes represented microwave power levels, while the suffixes indicated incubation weeks after microwave treatment. Specifically, CK0, 2 kW0, 4 kW0, 6 kW0 and 8 kW0 stood for soil samples collected at 0 days of incubation immediately after microwave treatment; CK15, 2 kW15, 4 kW15, 6 kW15 and 8 kW15 represented samples gathered after 15 weeks of incubation; CK30, 2 kW30, 4 kW30, 6 kW30 and 8 kW30 denoted samples harvested at 30 weeks of incubation.

### 2.3. Structural Characterization

An X-ray diffractometer (XRD, Ultima IV, Rigaku, Akishima, Japan) was used to analyze the mineral crystalline composition of soil. The test parameters were set as a Co target, a wide-angle scanning range of 5~90°, and a scanning speed of 10°/min. A Fourier transform infrared spectrometer (FTIR, Bruker, Ettlingen, Germany) was applied to characterize the variations in functional groups in soil organic matter; samples were prepared by the potassium bromide (KBr) tablet pressing method for full-wavelength scanning measurement. A thermogravimetric analyzer (TGA, STA449F3, Netzsch, Selb, Germany) was adopted to investigate the thermal stability and thermal decomposition characteristics of soil organic matter. Under a nitrogen atmosphere, the temperature program was arranged from 30 °C to 800 °C with a heating rate of 10 °C/min [24]. Each treatment was measured three times. A scanning electron microscope (SEM, HITACHI SU8010, Hitachi, Tokyo, Japan) was utilized to observe the microscopic morphology and aggregate structure of soil. Prior to observation, samples were fixed on conductive adhesive and were subjected to gold-spraying pretreatment before testing [25].

### 2.4. Determination of Soil Physicochemical Properties

Soil suspensions with water (1:2.5 WV-1) were prepared to estimate soil pH and electrical conductivity (EC) using a pH-conductivity multiparameter meter (PHS-3C, INESA Scientific Instrument Co., Ltd., Shanghai, China) [26,27]. An elemental analyzer was used to measure total nitrogen (TN) in the soil extracts (Elementar, Frankfurt, Hanau, Germany). The Molybdenum Blue procedure was used to assess available phosphorus (AP) using hydrochloric acid and ammonium fluoride [28]. Available potassium (AK) was extracted using ammonium acetate and quantified using flame photometry [29]. Total potassium (TK) and total phosphorus (TP) levels were determined by first digesting the soil using the H2SO4-HClO4 procedure and then calculating the levels, as described for AP and AK. The alkaline hydrolyzable diffusion method [30] and the potassium dichromate external heating method [31] were used to determine available nitrogen (AN) and soil organic matter (SOM). Each treatment was measured three times.

### 2.5. DNA Extraction and High-Throughput Sequencing

Total genomic DNA was extracted from soil samples collected at 0, 15 and 30 weeks from each treatment using the TIANamp Soil DNA Kit (TIANGEN Biotech Co., Ltd., Beijing, China). Qualified genomic DNA was transported to Wekemo Technology Group Co., Ltd., Shenzhen, China, for shotgun metagenomic sequencing on the Illumina platform.

DNA libraries were constructed using the RapidPlus DNA Lib Prep Kit for Illumina (ABclonal, Woburn, MA, USA, Cat. No. RK20208). This study adopted untargeted shotgun metagenomic sequencing, and no target-specific amplicon PCR was conducted. Library amplification and library quantification by qPCR were carried out following the manufacturer’s standard protocols and the sequencing provider’s routine optimized internal workflow. Detailed proprietary primer sequences and complete PCR cycling parameters are commercially confidential and cannot be fully disclosed. Sequencing was performed on the Illumina platform with a paired-end 150 bp strategy.

Raw sequencing reads were quality-filtered with Trimmomatic, and host-derived sequences were removed using Bowtie2. FastQC was used to evaluate data quality. For taxonomic profiling, DIAMOND was used to align non-redundant protein sequences against the NCBI NR database, followed by taxonomic classification with BASTA. Kraken2 (with an in-house microbial database) combined with Bracken was employed to calculate the relative abundance of microbial communities.

Host-free clean reads were assembled into contigs using MEGAHIT. Open reading frames were predicted from contigs via Prodigal, and redundant gene sequences were clustered using MMseqs2. Salmon was applied to calculate gene abundance. The transeq module within EMBOSS was used to translate nucleotide sequences into protein sequences for functional annotation. Protein sequences were aligned against the EggNOG, CAZy and CARD databases using DIAMOND to retrieve functional information, including KEGG, GO and COG annotations. The abundance values of genes assigned to the same functional family were summed to generate functional abundance matrices.

Genes with |log_2_FC| ≥ 1 and *p* < 0.05 were screened as differentially abundant genes (DAGs), followed by KEGG pathway enrichment analysis. On the basis of taxonomic and functional abundance profiles, hierarchical clustering, PCoA, LEfSe and Dunn’s test were implemented to identify differential microbial taxa and metabolic pathways among treatment groups. All bioinformatic analyses were conducted on the Wekemo Bioinformatics Cloud Platform (https://bioincloud.tech/) (All the software and websites used for raw data processing mentioned above were accessed on 15 June 2026).

### 2.6. Statistical Analysis

The data are presented as mean ± standard deviation. Statistical analyses were conducted using SPSS version 22.0 (IBM Corp., Armonk, NY, USA). Prior to ANOVA, the normality of data distribution and homogeneity of variances were verified using the Shapiro–Wilk test and Levene’s test, respectively. One-way analysis of variance (ANOVA) followed by Duncan’s multiple range test was used to evaluate significant differences (*p* < 0.05). The analytical workflows covered α diversity indices (Chao1, Shannon, Simpson, Pielou), Bray–Curtis distance-based PCoA, LEfSe differential taxa screening (LDA threshold = 3.5), RDA redundancy analysis, Mantel test, identification of differentially expressed genes, and KEGG enrichment visualization. Other images were generated using Origin Pro 2025 and Microsoft PowerPoint 2021.

## 3. Results and Discussion

### 3.1. Effects of Microwave Treatment on Soil Temperature

Real-time soil temperature under five microwave power gradients was continuously monitored in this experiment to clarify the soil heating characteristics. Previous studies had confirmed that most soil pests, pathogenic fungi and bacteria could be inactivated at 60 °C; heating above 70 °C could almost completely eliminate soil fungi, bacteria and plant viruses [32,33,34]. In this study (Figure 1d), the average temperatures of treatments from 4 kW to 8 kW all exceeded 60 °C. Meanwhile, both the average and maximum soil temperatures rose steadily with the increase in microwave power, showing a significantly positive correlation between power and heating effect, which was consistent with the research of Hong et al. [35]. Taking the 2 kW treatment group as an example, its dynamic temperature curve within the 10 min treatment period was shown in Figure 1e: the soil temperature increased rapidly from 0 to 5 min, the heating rate slowed down obviously from 5 to 10 min, and the peak temperature was stably maintained at 85–89 °C in the later stage. This trend indicated that the heat absorption capacity of the tested soil reached saturation under 2 kW microwave power. Further extending the treatment duration would only lead to massive soil water loss and organic matter carbonization without additional temperature elevation, which was consistent with the findings reported by Liu [36]. The identical thermal saturation phenomenon was also observed in the 4 kW, 6 kW and 8 kW treatment groups (Video S1). Combined with infrared thermal imaging data, a fixed treatment duration of 10 min provided uniform and comprehensive heating of the soil. Taking microwave energy consumption into consideration to avoid unnecessary energy waste, the treatment time was uniformly set to 10 min in the formal microwave experiment [37]. The equipment supported continuous power adjustment ranging from 0 to 10 kW. To protect the device, all power levels were set below 10 kW, and five gradients of 0, 2, 4, 6 and 8 kW were thereby established. The obvious temperature discrepancies induced by different power gradients may provide a physical basis for the subsequent divergent shifts in soil physicochemical properties and microbial community composition across all treatment groups.

### 3.2. Effects of Microwave Treatments on Soil Structure

**Figure 2:**
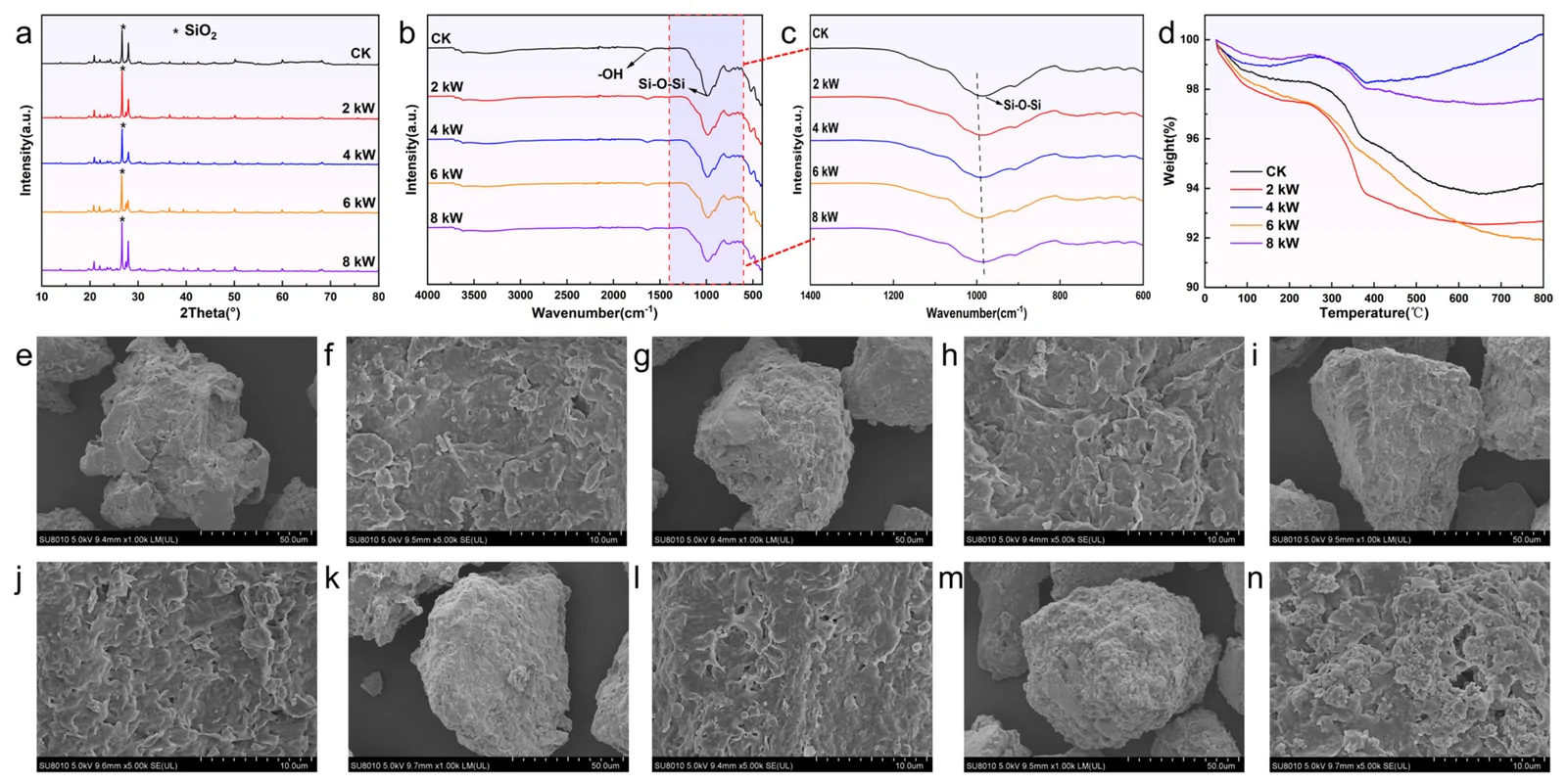
Effects of microwave treatments on soil structure. (**a**) XRD diffraction patterns; (**b**) full-range FTIR spectra; (**c**) local FTIR spectra at 1000–1100 cm^−1^; (**d**) thermogravimetric (TGA) curves; (**e**) and (**f**): SEM images of the CK group; (**g**) and (**h**): SEM images of the 2 kW treatment; (**i**) and (**j**): SEM images of the 4 kW treatment; (**k**) and (**l**): SEM images of the 6 kW treatment; (**m**) and (**n**): SEM images of the 8 kW treatment.

XRD patterns (Figure 2a) showed that no new crystalline phases were generated after microwave irradiation, and the primary mineral components of soil remained unchanged. The crystal phase transition temperature of natural silica exceeds 1200 °C [38], which is much higher than the maximum temperature generated during conventional microwave treatment. Therefore, microwave heating only introduces lattice defects without triggering mineral recrystallization and phase reconstruction. With the increase in microwave power and the corresponding rise in peak soil temperature, the characteristic diffraction peaks of SiO_2_ gradually broadened, dispersed and declined in relative intensity. Soil crystallinity decreased sequentially from the CK group to the 8 kW treatment, indicating that the stronger thermal effect induced by high temperature accumulated more lattice defects inside mineral particles and destroyed the ordered crystal structure.

No new characteristic absorption peaks were observed in the full-range Fourier transform infrared spectra (Figure 2b), confirming that microwave treatment does not trigger chemical reactions to generate new substances. Soil structural modification is mainly driven by elevated temperature, which induces physical rearrangement and dehydration condensation of surface hydroxyl groups. This process reduces the total content of -OH groups while increasing the relative proportion of Si-O-Si bonds, consistent with the XRD results. The characteristic Si-O-Si peak of SiO_2_ occurs at 1000–1100 cm^−1^ (Figure 2c). The peak of the CK group is narrow and sharp. As microwave power increases, the peak broadens and shifts slightly toward lower wavenumbers, revealing reduced long-range crystal ordering and accumulated microstructural defects within soil particles.

The thermal weight loss of soil is mainly derived from three processes: desorption of adsorbed water, decomposition of structural hydroxyl groups, and pyrolysis of trace organic matter [39]. As the microwave power increased from CK to 6 kW, the average soil temperature rose from 28.33 °C to 79.70 °C (Figure 1d); correspondingly, T5% decreased from 342.6 °C, Tmax declined from 481.3 °C to 428.4 °C, and the residual weight at 800 °C (W_800°C_) dropped from 94.2% to 91.9% (Table 1). These data indicated that medium and low microwave power destroyed the long-range ordered structure of SiO_2_, increased lattice defects, generated abundant micropores on particle surfaces and raised the specific surface area, thus reducing the overall thermal stability of soil and shifting thermal decomposition to lower temperatures. Nevertheless, T_5_%, T_max_ and W_800°C_ all rebounded under the 8 kW treatment (Figure 2d). This phenomenon could be attributed to the collapse and coalescence of partial micropores under ultrahigh thermal load, which reduced the specific surface area. Meanwhile, intensive dehydration and condensation of surface Si-OH groups reconstructed cross-linked Si-O-Si networks and restored the thermal stability of soil [40].

SEM was further applied to observe the microstructures of soil samples from the CK, 2 kW, 4 kW, 6 kW and 8 kW treatments (Figure 2: e and f represented the CK group; g and h represented the 2 kW group; i and j represented the 4 kW group; k and l represented the 6 kW group; m and n represented the 8 kW group). The results showed that microwave treatment did not alter the main skeleton structure of soil, but only modified the surface aggregate morphology and pore distribution, which was consistent with the XRD and FTIR data. Further SEM observations revealed that the gradient temperature differences generated by different microwave powers might directly differentiate the damage degree of soil aggregate structures. The CK group (Figure 2e,f) was dominated by dense massive sheet aggregates with neatly stacked clay layers and narrow interparticle voids; soil particles were tightly cemented by organic matter, with scarce micropores. The compact aggregate structure encapsulated abundant phosphorus and potassium nutrients [41], which might explain the low concentrations of available phosphorus (AP) and available potassium (AK) in CK soil, and the enclosed microenvironment also facilitated the enrichment of soil-borne pathogenic fungi [42]. The heating effect of the 2 kW treatment was relatively mild, with an average temperature of 45.77 °C (Figure 1d). Only slight dissociation of clay occurred, a small amount of surface organic films peeled off, and merely a few tiny micropores formed, while the overall aggregate framework remained intact (Figure 2g,h). This indicated that the thermal disturbance induced by low-power microwaves was weak and barely released occluded nutrients. The 4 kW treatment yielded an average temperature of 67.40 °C (Figure 1d), which triggered distinct changes in the original sheet-like mineral structures and drastically increased the quantity of micropores (Figure 2i,j). Aggregates dissociated and released encapsulated AP and AK; meanwhile, more active mineral sites were exposed to adsorb soluble salts and provide suitable habitats for microorganisms [43]. The average temperature further rose to 79.70 °C under 6 kW and reached 90.37 °C under 8 kW (Figure 1d). High-temperature microwave treatments at 6 kW (Figure 2k,l) and 8 kW (Figure 2m,n) further accelerated the dissociation of soil aggregates and released more trapped nutrients in the short term. However, high temperatures induced massive pyrolysis of soil organic matter and severe loss of organic cementing substances, which might weaken the nutrient retention capacity of soil and exert adverse effects on the colonization and recovery of microbial communities.

### 3.3. Effects of Microwave Treatments on Soil Physicochemical Properties

Soil total nutrients, available nutrients, soil organic matter (SOM), pH and electrical conductivity (EC) are core indicators reflecting soil fertility status, and their variations may be closely driven by structural changes in soil aggregates induced by microwave heating [44]. As shown in Table 2, significant differences (*p* < 0.05) were observed in all soil physicochemical indicators under various microwave treatments. With the increase in microwave power and incubation time, soil TN, TP, TK and SOM generally decreased gradually; AN, AP and AK rose first and then declined slightly in the later stage; soil pH decreased slowly overall, while EC presented a trend of decreasing first and then increasing.

Compared with the CK group, the variation ranges of all indicators under the 2 kW microwave treatment were small, indicating that the disturbance induced by microwave at this power was limited, and most indicators showed no significant differences (*p* < 0.05). The average temperature of the 4 kW microwave treatment reached 67.40 °C, which effectively broke partial soil aggregate structures and decomposed part of bound organic matter in soil, thereby promoting the mineralization and consumption of soil TN, TP and TK. The decrease in AN content might be related to the volatilization and loss of ammonium nitrogen [45]. Meanwhile, large amounts of bound phosphorus and potassium that were hardly available to crops were released and converted into AP and AK, realizing the activation of soil potential nutrients, which was consistent with the research results of Li et al. [19]. A small amount of low-molecular organic acids produced during organic matter decomposition might partially account for the slight decline in soil pH, and moderate acidification alleviated the tendency of soil salinization [46]. EC remained at a low level under this treatment, which might be attributed to the absorption and utilization of soluble salt ions by surviving soil microorganisms. The concentration of free ions was effectively regulated, reducing the risk of secondary salinization caused by salt accumulation [47,48].

The average temperatures of the 6 kW and 8 kW microwave treatments reached 79.70 °C and 90.37 °C, respectively. The stronger thermal effects at high temperatures greatly reduced the contents of soil TN, TP, TK, AN and SOM, and nutrient loss intensified as the incubation time extended. Although microwaves with excessively high power released abundant AP and AK in the short term, the contents of AP and AK decreased significantly, pH dropped sharply, and EC rose drastically after 30 weeks of soil incubation. The above results suggested that although short-term high-intensity microwave heating promoted nutrient release temporarily, it might bring adverse effects in the long run. Higher microwave power generated greater temperature rises in soil, accelerated the decomposition of soil organic matter, and released large quantities of aggregate-bound nitrogen, phosphorus and potassium. Meanwhile, AN volatilized in the form of ammonia gas, directly leading to massive losses of TN and AN. Continuous mineralization of SOM caused constant consumption of soil phosphorus and potassium, which was consistent with the report of Yao et al. [49] In addition, drastic thermal decomposition of organic matter might produce abundant organic acids. Combined with the bactericidal effect of high temperature, the acid-base buffering capacity of soil declined, and soil acidification was significantly aggravated [50]. EC increased rapidly during the 15–30 week incubation period. This phenomenon might result from massive microbial death; surviving microorganisms exhibited weakened capacity to absorb and utilize ions, leading to continuous accumulation of free salt ions and gradual elevation of EC, which further triggered the risk of secondary salinization [51].

### 3.4. Effects of Microwave Treatments on Soil Microbial Alpha Diversity

Microbial alpha diversity indices could directly reflect the stability of soil microecology, among which Chao1, Simpson, Shannon and Pielou indices represented species richness, community dominance, overall diversity and species evenness, respectively [52]. As shown in Table 3, the bacterial Chao1 indices of all microwave-treated soils were higher than those of the CK group at the same incubation stage, and generally increased with the rise in microwave power. The bacterial Chao1 indices of the 6 kW0 and 8 kW0 groups reached the maximum values among all treatments. Meanwhile, the bacterial Shannon and Pielou indices of the 8 kW group were significantly higher than those of other groups. These results indicated that microwave irradiation improved the diversity, richness and evenness of bacterial communities.

In general, the thermal effect of microwaves killed a large number of microorganisms. However, most microorganisms in continuously cropped soils resided inside soil aggregates or stayed in a dormant state. High-energy microwave oscillations broke partial soil aggregates, and thermal effects released dormant heat-resistant bacteria encapsulated by cementing substances. After numerous heat-sensitive bacteria were eliminated by high temperature, heat-resistant groups such as bacilli were exposed and proliferated rapidly, which might account for the significant increase in bacterial species richness [19,53]. Microwave treatment at 4 kW moderately activated soil AP and AK, which provided available nutrients for heat-resistant functional bacteria and further raised community diversity [54]. As incubation time extended to 15 and 30 weeks, the bacterial Chao1 and diversity indices of all microwave groups declined continuously, and the species richness of the 8 kW30 group decreased significantly. This phenomenon might be directly related to the rapid consumption of soil organic matter, insufficient nitrogen supply and decreased pH induced by high-power microwaves. Nutrient scarcity restricted the reproduction of most bacteria and gradually reduced community richness, which corresponded to the detection results of soil physicochemical properties and was consistent with the findings reported by Orozco-Mosqueda [55].

The variation patterns of fungal communities differed markedly from those of bacterial communities (Table 3). The fungal Chao1, Shannon and Pielou indices were the highest in the 2 kW0 group, which indicated the greatest fungal diversity and richness. However, fungal richness showed no superiority in the 6 kW0 and 8 kW0 groups and decreased significantly relative to the CK group. This might imply that fungi were more sensitive to high-temperature stress. Under low-power (2 kW) microwave irradiation, the thermal effect failed to eliminate fungi on a large scale, while high temperatures generated by 4 kW, 6 kW and 8 kW microwaves directly killed a great many fungi and drastically reduced the number of fungal species. Unlike the continuous declining trend of the fungal Chao1 index in the CK group with prolonged incubation time, the Chao1 indices of all microwave-treated groups decreased first and then increased. After 30 weeks of incubation, the indices related to fungal richness and diversity in each microwave treatment group were higher than those of CK30. The above results illustrated that high microwave temperature exerted a strong influence on fungal communities at the initial treatment stage, whereas fungal communities could recover gradually after incubation, and the richness and diversity of fungal communities at 30 weeks were superior to those of untreated soil (CK30). These findings might partially demonstrate the application potential of microwave treatment. Nevertheless, the drastic thermal effect induced by high-power (8 kW) microwaves caused severe damage to fungal communities, and the recovery process of fungal populations remained uncertain. A potential risk of disrupting the original soil fungal microecological balance might exist in the long term [56].

### 3.5. Effects of Microwave Treatments on Microbial Beta Diversity

To further distinguish the overall differences in microbial community composition under different microwave treatments and incubation durations, a Bray–Curtis distance matrix was constructed based on species abundance, and principal coordinate analysis (PCoA) of microbial communities was performed. By characterizing microbial beta diversity, this method visualized the distribution patterns of samples from different treatments in coordinate space and directly reflected the dissimilarities in community structural similarity [57].

**Figure 3:**
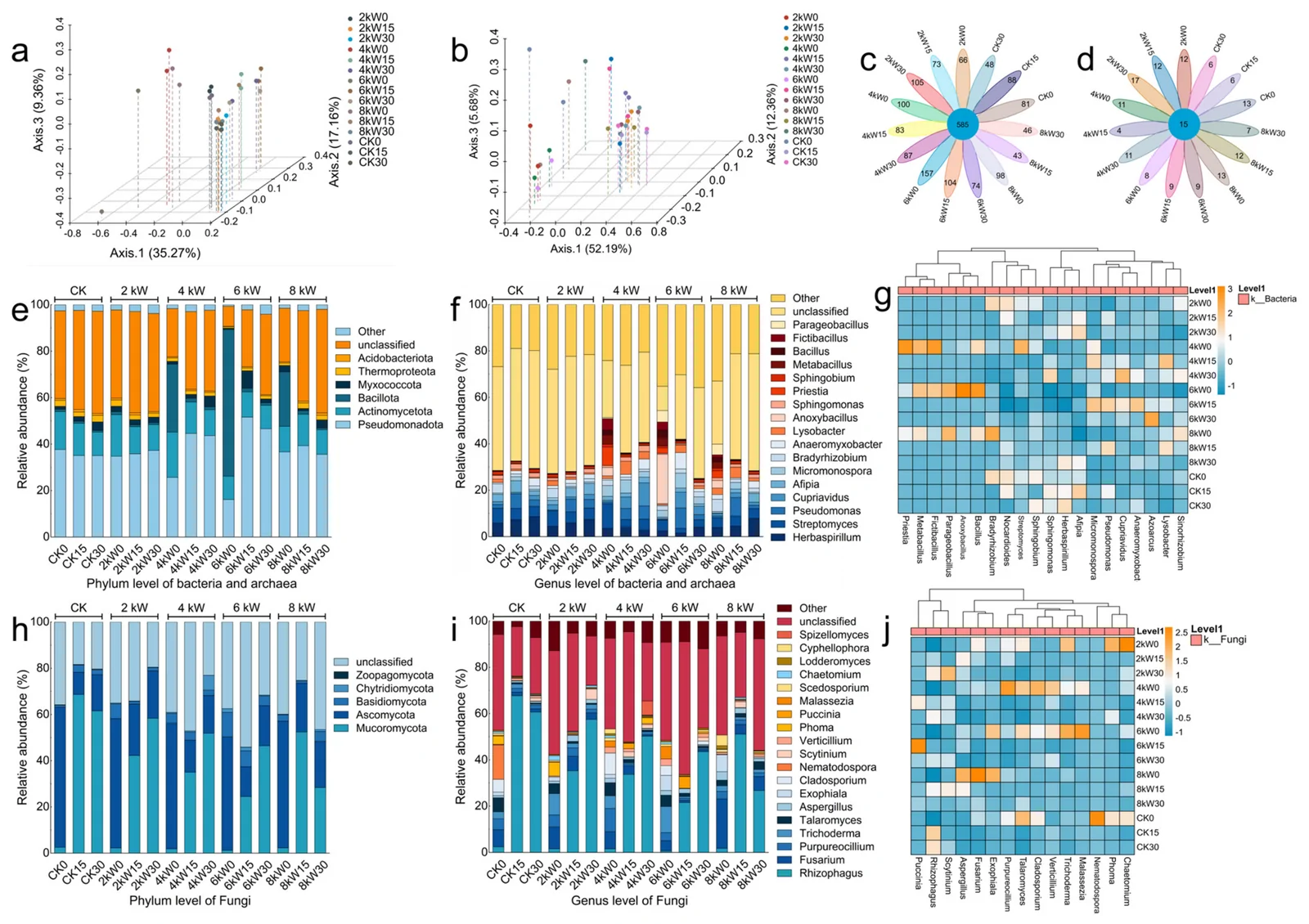
Effects of microwave treatments on microbial beta diversity and species composition. (**a**) PCoA of bacterial communities; (**b**) PCoA of fungal communities; (**c**) petal diagram of bacteria; (**d**) petal diagram of fungi; (**e**) relative abundances of bacteria and archaea at the phylum level; (**f**) relative abundances of bacteria and archaea at the genus level; (**g**) cluster heatmap of the top 20 bacterial genera with the highest relative abundances; (**h**) relative abundances of fungi at the phylum level; (**i**) relative abundances of fungi at the genus level; (**j**) cluster heatmap of the top 15 fungal genera with the highest relative abundances. Specifically, CK0, 2 kW0, 4 kW0, 6 kW0 and 8 kW0 stood for soil samples collected at 0 days of incubation immediately after microwave treatment; CK15, 2 kW15, 4 kW15, 6 kW15 and 8 kW15 represented samples gathered after 15 weeks of incubation; CK30, 2 kW30, 4 kW30, 6 kW30 and 8 kW30 denoted samples harvested at 30 weeks of incubation.

Figure 3a,b presented the 3D-PCoA plots of bacterial and fungal communities, respectively. As illustrated in Figure 3a,b, Axis 1 explained 35.27% of the total variation for bacteria and 52.19% for fungi, which indicated that bacterial and fungal communities were mainly shaped by the gradient of microwave power. For bacteria (Figure 3a), the 6 kW and 8 kW groups were distinctly separated from the 2 kW, 4 kW and CK groups in the PCoA space. For fungi (Figure 3b), all microwave-treated groups were clearly differentiated from the CK group, and the fungal community structures of the 6 kW and 8 kW groups exhibited the largest discrepancies relative to the CK group, whereas incubation time exerted minor effects on both bacterial and fungal community structures. The sample distribution in Figure 3a,b showed obvious separation between microwave treatments and the CK group, which suggested that microwave thermal effects thoroughly reshaped the overall composition of indigenous soil microbial communities. Samples treated at 2 kW and 4 kW clustered closely with high similarity in community structure, while samples of the 6 kW and 8 kW groups aggregated in an independent region and were clearly isolated from the 2 kW and 4 kW groups.

These results indicated that the magnitude of microwave power was positively correlated with the differentiation of microbial community composition. Instant high temperature induced by microwaves might impose strong environmental stress. Thermal intensity under 2 kW and 4 kW treatments was moderate, which only suppressed some microorganisms and consumed limited soil organic matter and available nutrients, thereby retaining most dominant microbial taxa. In contrast, extreme high temperatures generated by 6 kW and 8 kW microwaves eliminated a large number of dominant microbes. Combined with multiple environmental stresses induced by thermal disturbance, the overall structure of microbial communities was significantly altered, which was consistent with the findings reported by Gai [58].

### 3.6. Effects of Microwave Treatments on Microbial Community Composition

Petal diagrams visually displayed the differences in the number of bacterial and fungal species among different treatment groups (Figure 3c,d). There were 585 shared bacterial species and 15 shared fungal species between the microwave-treated groups and the CK group, indicating that these species possessed strong thermal stability. The number of unique microbial species differed significantly, and soils treated at 4 kW and 6 kW contained relatively more unique microbial taxa sensitive to environmental changes.

Statistical analyses were further conducted on the relative abundances of soil microorganisms at the phylum and genus levels after incubation for 0, 15 and 30 weeks under different microwave powers, and cluster heatmaps were combined to distinguish differential microbial taxa. Figure 3e showed the relative abundances of bacteria and archaea at the phylum level. *Pseudomonadota* and *Actinomycetota* dominated the untreated CK soil, while *Bacillota* became an additional dominant phylum in all microwave-treated samples. The relative abundance of Bacillota increased significantly under 4–8 kW treatments, and then dropped to the CK level after 30 weeks of incubation. Medium and high microwave powers suppressed *Pseudomonadota* and *Actinomycetota*, and these two phyla recovered slowly as incubation proceeded.

These phenomena indicated that most taxa of *Pseudomonadota* and *Actinomycetota* were mesophilic soil microorganisms that decomposed organic residues and synthesized antibacterial substances to suppress soil pathogens, which explained their sensitivity to high temperatures. By contrast, *Bacillota* could form endospores to resist extreme environments and multiplied rapidly by utilizing phosphorus and potassium nutrients on the spot, yet their abundance declined gradually in the later stage, accompanied by massive consumption of nutrients that led to nutrient shortage in the late incubation period, which was consistent with the research results of Khan [59]. The results at the genus level (Figure 3f) further demonstrated that the abundances of spore-forming genera such as *Bacillus*, *Metabacillus*, *Fictibacillus* and *Parageobacillus* rose significantly increased, which matched the results at the phylum level. Treatments of 2 kW and 4 kW exerted slight or even positive effects on the relative abundances of growth-promoting and disease-resistant genera (*Bradyrhizobium*, *Sphingomonas*, *Streptomyces* and *Lysobacter*). *Bradyrhizobium* drove biological nitrogen fixation to supply nitrogen for sugarcane [60]; *Streptomyces* and Lysobacter inhibited pathogenic fungi and nematodes and jointly improved soil disease resistance to facilitate sugarcane growth [61]. However, obvious inhibitory effects occurred under microwave irradiation at 6 kW and 8 kW. Heatmap clustering (Figure 3g) revealed that the 2 kW group showed no obvious distinction from the CK group, while microwave treatments of 4–8 kW produced significant differences, and genera including *Priestia*, *Fictibacillus* and *Anoxybacillus* were significantly upregulated.

Figure 3h presented the relative abundances of fungi at the phylum level. *Ascomycota*, *Mucoromycota* and *Basidiomycota* constituted the dominant fungal phyla. Microwave treatments reduced the abundance of *Ascomycota* and raised the abundance of *Basidiomycota*, but exerted little influence on *Mucoromycota*. This might be attributed to the lack of heat-resistant endospores in fungi, which made their cell membranes prone to rupture and hyphae easy to deactivate under high temperatures; excessive decomposition of organic matter caused by heating also restrained the subsequent recovery of fungi [62]. The variation trend of *Rhizophagus* abundance was highly consistent with that of *Mucoromycota* (Figure 3i), which implied a strong correlation between them. *Rhizophagus* extended extraradical hyphae and secreted organic acids and phosphatases to dissolve mineral-fixed and insoluble organic phosphorus, thereby greatly improving the supply efficiency of soil available phosphorus and potassium [63,64]. The relative abundances of *Trichoderma* and *Purpureocillium* increased significantly under treatments of 2–6 kW, and these microorganisms exerted beneficial effects on sugarcane growth. *Trichoderma* secreted multiple cellulases and ligninases to decompose inert soil organic matter and simultaneously released organically bound nitrogen and phosphorus [65]. *Purpureocillium* secreted highly active chitinases and proteases to degrade the chitin structures on nematode eggshells and body surfaces and sharply reduced the quantity of root-knot nematodes in soil [66]. Nevertheless, these two genera were obviously suppressed under the microwave treatment of 8 kW. Clustering analysis (Figure 3j) showed that microwave irradiation greatly altered the composition and abundance of fungal communities, and fungi such as *Purpureocillium* and *Trichoderma* were upregulated. The enrichment effect weakened with the extension of incubation time and resulted in minor discrepancies among community structures, which meant that fungal communities recovered gradually in the later incubation stage, and Ferriss [67] reported similar phenomena.

### 3.7. Differential Analysis of Microbial Species

**Figure 4:**
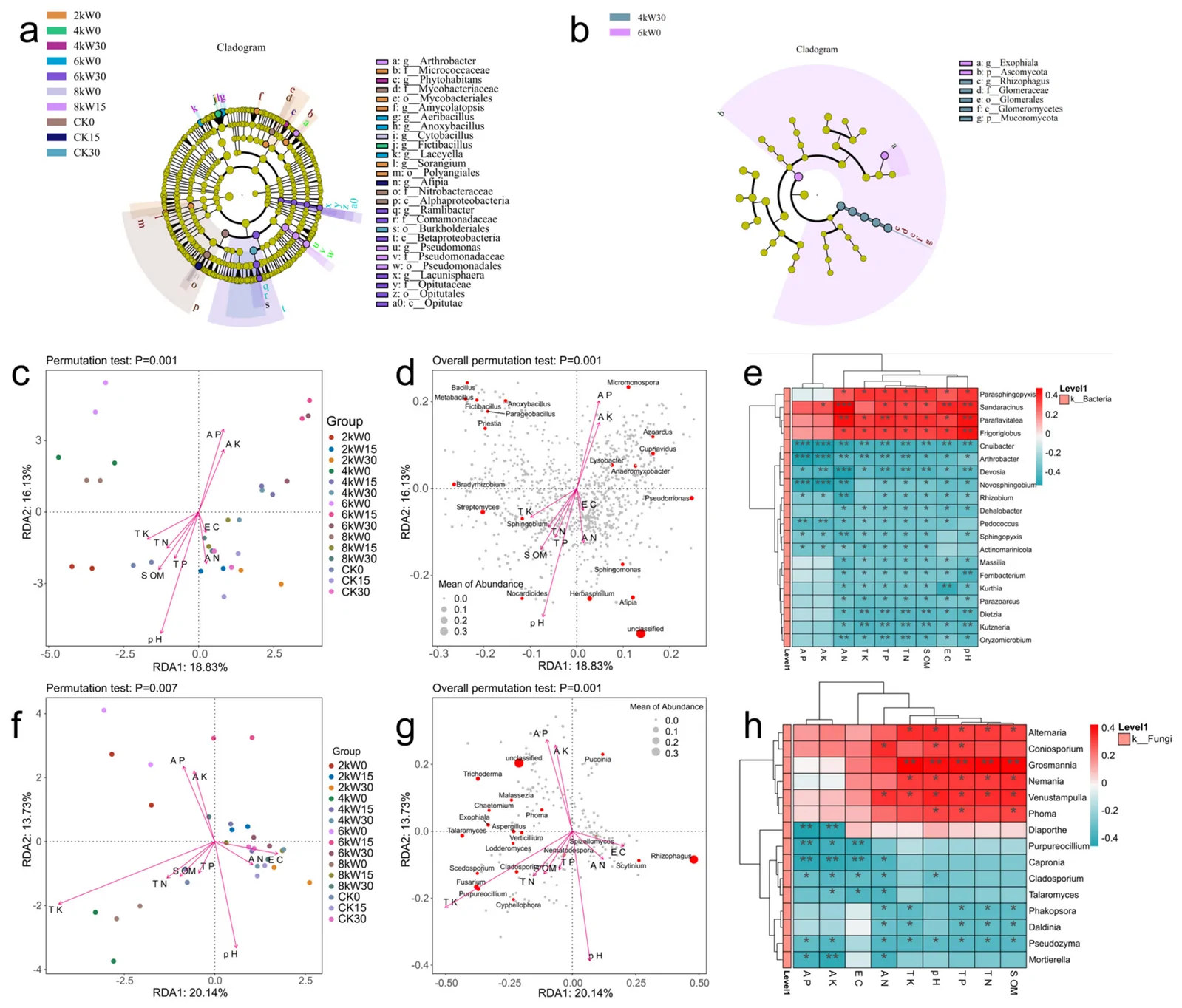
Differential species analysis and correlation analysis under different microwave treatments. (**a**) LEfSe cladogram of bacterial communities; (**b**) LEfSe cladogram of fungal communities; (**c**) RDA ordination plot showing the relationships between bacterial communities and soil environmental factors; (**d**) ordination plot revealing the correlations between dominant bacterial genera and environmental factors; (**e**) correlation heatmap between bacterial genera and environmental factors; (**f**) RDA ordination plot showing the relationships between fungal communities and soil environmental factors; (**g**) ordination plot revealing the correlations between dominant fungal genera and environmental factors; (**h**) correlation heatmap between fungal genera and environmental factors. Specifically, CK0, 2 kW0, 4 kW0, 6 kW0 and 8 kW0 stood for soil samples collected at 0 days of incubation immediately after microwave treatment; CK15, 2 kW15, 4 kW15, 6 kW15 and 8 kW15 represented samples gathered after 15 weeks of incubation; CK30, 2 kW30, 4 kW30, 6 kW30 and 8 kW30 denoted samples harvested at 30 weeks of incubation. * *p* < 0.05, ** *p* < 0.01, *** *p* < 0.001, compared with the control group.

Figure 4a,b showed the LEfSe cladograms of bacteria and fungi, respectively. The results directly displayed the biomarker taxa with significant differences at various microbial taxonomic levels in samples treated with different microwave powers and incubated for different durations [68]. LEfSe analysis (LDA threshold = 3.5) revealed that different microwave treatments significantly altered microbial community structures at the genus level (*p* < 0.05).

The LEfSe results for bacteria (Figure 4a) indicated that the significantly enriched taxa were divided into two major functional groups, which were clearly differentiated among CK, medium-low power (2 kW, 4 kW) and high power (6 kW, 8 kW) samples. *Firmicutes* genera including *Aeribacillus*, *Anoxybacillus*, *Fictibacillus*, *Laceyella* and *Cytobacillus* were significantly enriched in the 6 kW and 8 kW microwave samples (8 kW0, 6 kW0, 6 kW30). These bacillus-type microorganisms formed endospores and tolerated high temperatures and oligotrophic conditions. *Actinobacterial* taxa such as *Arthrobacter*, *Amycolatopsis*, *Phytohabitans* and *Mycobacteriaceae*, as well as myxobacterial *Sorangium* and *Polyangiales*, mainly accumulated in CK, 2 kW and 4 kW samples. These microbes degraded recalcitrant soil organic matter and secreted antimicrobial compounds to inhibit soil-borne pathogens, yet they exhibited low tolerance to high-temperature stress [69]. *Afipia*, *Ramlibacter*, *Pseudomonas* and *Comamonadaceae* mostly appeared in CK and 4 kW groups. *Pseudomonas* solubilized insoluble phosphorus and produced auxins to stimulate sugarcane root growth. Thermal stress generated by 4 kW microwave did not eliminate large quantities of these microorganisms, and balanced soil carbon and nitrogen nutrients supported their colonization. In contrast, high temperatures induced by high-power irradiation, combined with subsequent soil acidification and salt accumulation, drastically reduced the relative abundance of these taxa [70]. In terms of sample community distribution characteristics, CK, 2 kW and 4 kW samples harbored abundant clades of growth-promoting and organic matter-degrading bacteria and exhibited high community similarity. By contrast, samples under 6 kW and 8 kW treatments only enriched heat-resistant spore-forming bacteria, accompanied by massive losses of indigenous dominant actinobacteria and Pseudomonas. Such microbial community composition delivered short-term nutrient release benefits, yet it might impair long-term nutrient cycling and disease suppression capacity, which was unfavorable for continuous sugarcane cultivation.

The fungal LEfSe cladogram (Figure 4b) identified fungal taxonomic units with significant differences across all treatments, which mainly comprised *Ascomycota*, *Mucoromycota* and *Glomeromycetes*. Among them, *Rhizophagus* belonging to *Glomeraceae* and *Glomerales* served as the core beneficial differential taxon and was significantly and abundantly enriched in the 4 kW30 samples. *Ascomycota* and *Mucoromycota* were distributed more widely in the CK group, and their community structures were reshaped after microwave irradiation. Treatments at 2 kW and 4 kW facilitated the enrichment of beneficial taxa such as *Glomeromycota*, whereas 6 kW and 8 kW treatments damaged and inhibited *Glomeromycota* communities, leaving only a small number of stress-tolerant saprophytic fungi. *Exophiala*, a stress-resistant fungus affiliated with *Ascomycota*, was specifically enriched in the 6 kW0 samples [71]. As an arbuscular mycorrhizal fungus, *Rhizophagus* formed symbiosis with crop roots to activate soil phosphorus and potassium and stabilize soil aggregates, and it antagonized pathogenic fungi simultaneously [72]. The thermal intensity of 4 kW microwave exerted minor impacts on the hyphae and spores of *Rhizophagus*. As the incubation time extended to 30 weeks, the soil environment became stable, and this fungus proliferated massively and turned into a characteristic differential taxon. In contrast, short-term high temperature under 6 kW directly killed most symbiotic beneficial fungi, and only a small number of saprophytic fungi tolerant to extremely high temperature, such as *Exophiala*, survived, which might deprive the microbial community of the long-term growth-promoting functions derived from mycorrhizal symbiosis.

### 3.8. Correlation Analysis

Redundancy analysis (RDA) was performed for bacterial and fungal communities based on soil physicochemical properties, pH, EC and other indicators to analyze the effects of soil physicochemical variables on microbial community composition. RDA visually revealed the correlations between variations in microbial community structure and soil environmental factors, quantified the explanation rate of each physicochemical indicator for community spatial differentiation, and identified the key environmental gradients driving microbial succession [73].

Figure 4c showed the RDA ordination plot of bacterial communities and soil environmental factors, with RDA1 explaining 18.83% of total variation and RDA2 explaining 16.13%. The vectors of SOM, TN, TP, AN and pH pointed toward the negative axis of RDA1 in the same direction, while AP, AK and EC were distributed along the positive axis of RDA1, forming an obvious negative gradient. All samples from the CK and 2 kW groups concentrated in the negative region characterized by high SOM and neutral pH, and samples from the 4 kW group located in the middle area, indicating that soil physicochemical indicators probably fell within a relatively balanced range. All samples of the 6 kW and 8 kW groups aggregated in the positive region with high available nutrients, high EC and low pH; moreover, samples under the same power continuously shifted toward the positive direction of RDA1 as incubation time elapsed. The above results indicated that continuous SOM depletion, salt accumulation and soil acidification (reduced pH) throughout the entire incubation period jointly reshaped bacterial microhabitats, and these processes constituted the core driving forces underlying microenvironmental transformation.

Figure 4d presented the ordination plot illustrating correlations between dominant bacterial genera and environmental factors. Spore-forming genera including *Bacillus*, *Fictibacillus* and *Parageobacillus* were significantly positively correlated with AP, AK, EC and low pH, corresponding to the extreme environments under 6 kW and 8 kW high-power treatments. In contrast, *Bradyrhizobium*, *Streptomyces*, *Sphingomonas* and *Lysobacter* were strongly associated with SOM, TN and neutral pH, and mainly enriched in CK, 2 kW and 4 kW treatments. Correlation markers between various microbial taxa and soil physicochemical factors demonstrated that most bacteria exhibited extremely significant positive correlations with SOM and TN, whereas heat-resistant stress-tolerant bacilli adapted to acidified and high-salinity environments [74].

The correlation heatmap between bacteria and environmental factors (Figure 4e) further verified that *Actinobacteria* and *Rhizobial* taxa such as *Arthrobacter*, *Rhizobium*, *Sphingopyxis*, *Dietzia* and *Kutzneria* had significant positive correlations with SOM, TN, TP, AN and pH. Thermotolerant spore-forming bacteria including *Parageobacillus* and *Bacillus* showed significant positive correlations with AP, AK and EC. These findings indicated that organic matter and suitable pH constituted essential conditions for the survival of soil bacteria, and high-salinity and acidic environments only selected single stress-resistant spore-forming bacterial communities, which was consistent with the report published by Hirte [75].

Figure 4f displayed the RDA ordination plot of fungal communities and soil environmental factors, in which RDA1 accounted for 20.14% of total variation, and RDA2 accounted for 13.73%. Fungal communities were more sensitive to environmental fluctuations than bacterial communities. Samples of CK, 2 kW and 4 kW clustered closely on the negative side of RDA1, while samples of 6 kW and 8 kW were independently distributed in the positive region with greater separation between groups. As incubation time increased, samples from the 6 kW and 8 kW groups continuously moved toward stressful conditions with high EC and low pH, and fungal communities gradually succeeded toward stress-resistant taxa [76].

Figure 4g was the ordination plot reflecting correlations between dominant fungal genera and environmental factors. Beneficial fungi, including *Trichoderma*, *Rhizophagus* [77], *Purpureocillium* and *Talaromyces* [78], were distributed in the same direction as SOM, TN and appropriate pH, matching the balanced soil environment of the 4 kW treatment. Soil-borne pathogens such as *Fusarium* [79], *Verticillium* [80], *Alternaria* [81] and *Phoma* [82] were significantly correlated with high EC and acidic conditions, and presented higher abundances under 6 kW and 8 kW treatments.

The correlation heatmap between fungi and environmental factors (Figure 4h) revealed that *Mortierella*, *Talaromyces*, *Purpureocillium*, *Rhizophagus* and *Trichoderma* all had significant positive correlations with SOM, TN, TP, AN and neutral pH. By contrast, pathogenic fungi including *Alternaria*, *Diaporthe* and *Phoma* were significantly positively correlated with EC and low pH. These results illustrated that SOM depletion, soil acidification and salinization induced by high-power microwave treatments greatly suppressed mycorrhizal and biocontrol fungi, thereby creating favorable conditions for the proliferation of pathogenic fungi.

Taken together, fungal communities were more sensitive to environmental shifts than bacterial communities. This could be attributed to the fact that certain taxa within *Bacillota* can form thermotolerant endospores to withstand extremely high temperatures induced by microwave irradiation, whereas most fungi exhibit weaker heat resistance compared with spore-forming bacteria [83,84]. Appropriate levels of SOM, TN and neutral pH facilitated the proliferation of beneficial microorganisms. In contrast, environments characterized by depleted SOM, high EC and low pH favored the enrichment of Bacillus and pathogenic fungi, which further suppressed the growth of other fungal groups. Treatments with 6 kW and 8 kW microwaves triggered severe SOM loss and intensified soil acidification (reduced pH), thereby hindering the healthy development of microbial communities. By comparison, the 4 kW microwave treatment achieved a more balanced soil microenvironment and well-structured microbial community.

### 3.9. Differential Gene Analysis

With the CK group as the control, volcano plots of differentially expressed genes and KEGG enrichment bubble plots were constructed for comparisons between the CK group and microwave treatments of 2 kW, 4 kW, 6 kW and 8 kW, respectively. Volcano plots distinguished significantly upregulated genes, significantly downregulated genes and non-differentially expressed genes, while bubble plots illustrated metabolic, signaling and resistance pathways significantly enriched by differentially expressed genes.

**Figure 5:**
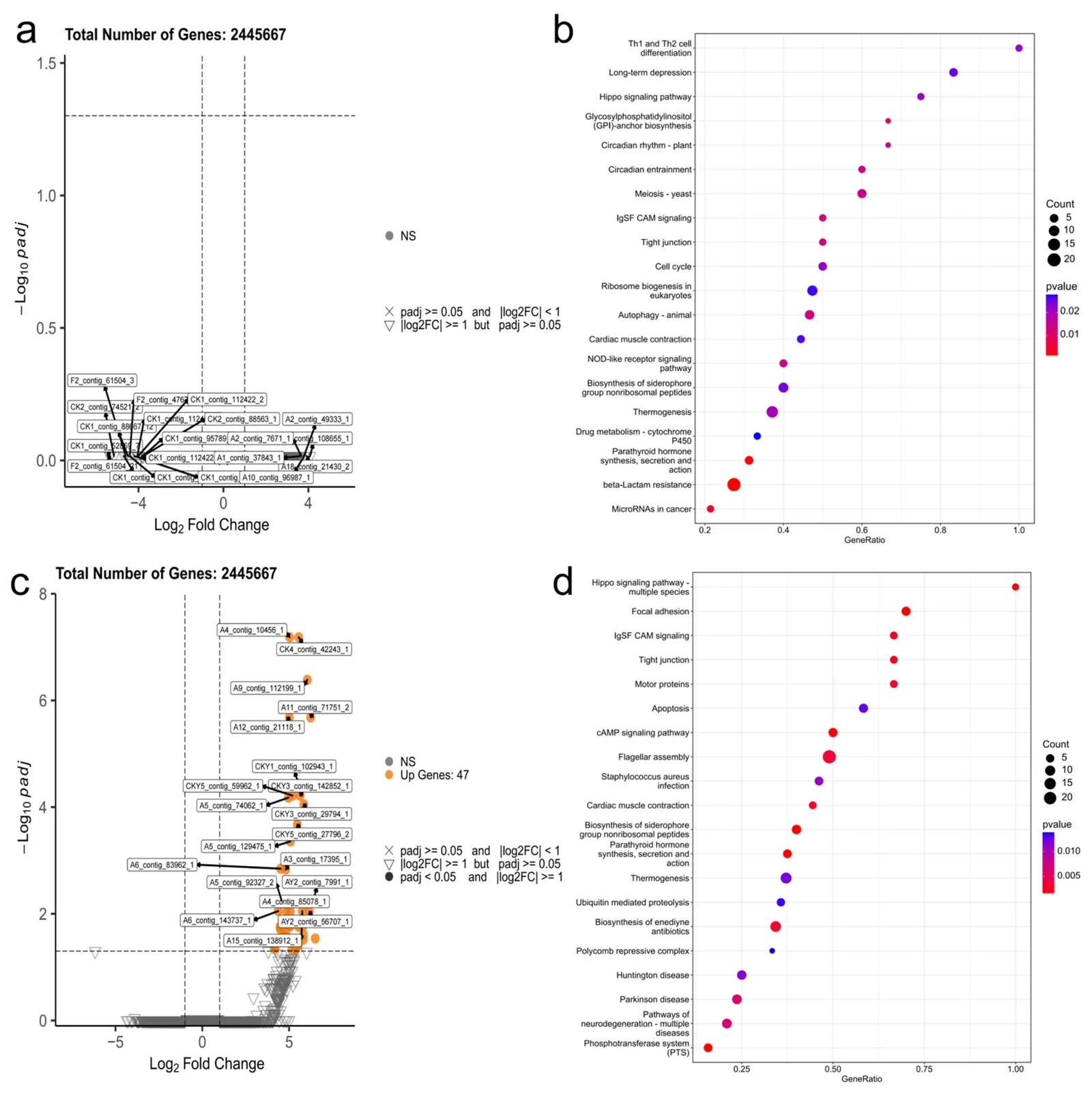
Volcano plots of differentially expressed genes and KEGG enrichment bubble diagrams under different microwave treatments. (**a**) Volcano plot of differentially expressed genes between the 2 kW treatment and CK; (**b**) KEGG enrichment bubble diagram for the 2 kW treatment; (**c**) volcano plot of differentially expressed genes between the 4 kW treatment and CK; (**d**) KEGG enrichment bubble diagram for the 4 kW treatment. In the volcano plot, gray dots represent genes with no significant change, and orange dots denote significantly up‑regulated genes. Symbols at the bottom‑right corner indicate the significance levels.

**Figure 6:**
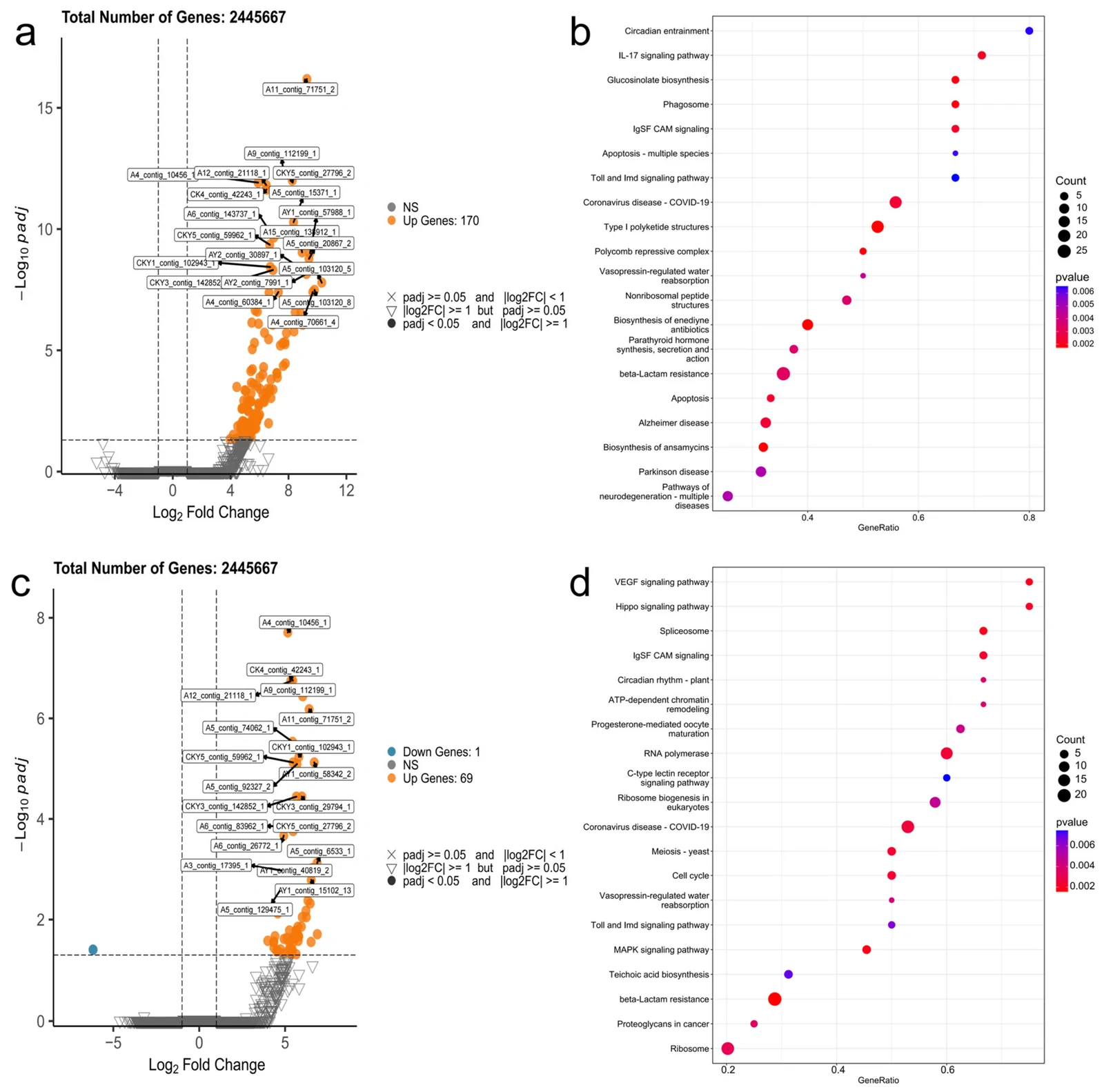
Volcano plots of differentially expressed genes and KEGG enrichment bubble diagrams under different microwave treatments. (**a**) Volcano plot of differentially expressed genes between the 6 kW treatment and CK; (**b**) KEGG enrichment bubble diagram for the 6 kW treatment; (**c**) volcano plot of differentially expressed genes between the 8 kW treatment and CK; (**d**) KEGG enrichment bubble diagram for the 8 kW treatment. In the volcano plot, gray dots represent genes with no significant change, and orange dots denote significantly up‑regulated genes. Symbols at the bottom‑right corner indicate the significance levels.

The results in Figure 5a showed that only a small number of genes exhibited significantly differential expression in the 2 kW group relative to the CK group, with extremely few significantly upregulated and downregulated genes and the vast majority of genes showing no significant differences. The KEGG enrichment results (Figure 5b) presented low enrichment levels, and only a small number of basic pathways related to heat tolerance and preliminary bacteriostasis were enriched. These findings indicated that the thermal interference intensity of microwave treatment at 2 kW was weak, which only slightly stimulated basic stress-resistant metabolism in microorganisms, exerted limited influence on the overall gene transcription level of soil microorganisms, and did not trigger large-scale reconstruction of microbial metabolic and functional pathways. The volcano plot of the 4 kW group (Figure 5c) identified 69 significantly upregulated genes and a small quantity of downregulated genes. The enriched pathways in the corresponding KEGG analysis (Figure 5d) mainly included heat response, cell cycle, resistance repair, nutrient transformation and secondary metabolite synthesis pathways with moderate and stable enrichment degrees, suggesting that microwave treatment at 4 kW might activate the growth-promoting and bacteriostatic functions of partial microorganisms at the molecular level. A total of 170 significantly upregulated genes were detected in the 6 kW group (Figure 6a), and the enrichment degree of KEGG pathways (Figure 6b) increased markedly, with abundant enrichment of biological control and nutrient cycling pathways such as siderophore synthesis, nonribosomal peptide synthesis, nutrient transformation and immune regulation [85,86,87]. This indicated that the metabolic potential of microorganisms for activating soil nutrients was fully stimulated after 6 kW microwave treatment. Nevertheless, short-term enrichment of these high-energy-consuming secondary metabolic pathways might lead to excessive consumption of soil SOM and nutrient loss in the later stage, which was unfavorable for maintaining the long-term stability of soil microbial communities and consistent with the detection results of soil physicochemical properties. The number of significantly upregulated genes decreased drastically in the 8 kW group (Figure 6c), and the corresponding KEGG analysis (Figure 6d) only enriched pathways involved in chromatin remodeling, heat tolerance and cellular damage repair, whereas almost no enrichment was observed for symbiosis-related and nutrient activation pathways. This phenomenon might be attributed to the elimination of a large number of beneficial microbial populations induced by extremely high temperature under microwave irradiation, so that microbial metabolism merely sustained basic survival and stress response functions.

## 4. Conclusions

This study adopted a 915 MHz continuous industrial microwave device with five power gradients (0, 2, 4, 6, 8 kW) and a fixed treatment duration of 10 min. Combined with incubation experiments lasting 0, 15 and 30 weeks, this study investigated the effects of gradient microwave heating on the structure, physicochemical properties and microbial characteristics of sugarcane continuous cropping soil.

The microwave thermal effect intensified with increasing power. Microwave treatment did not generate new mineral crystalline phases, but altered the microstructure of aggregates and released occluded soil nutrients such as phosphorus and potassium. Thermal imaging results demonstrated that the 915 MHz industrial microwave could penetrate soil layers and achieve uniform soil heating within 0–10 min of microwave treatment. Temperature was positively correlated with microwave power; the higher the microwave power, the faster the soil temperature rose.

The 4 kW microwave treatment effectively disintegrated compact soil aggregates, alleviated soil acidification and secondary salinization, and avoided excessive SOM consumption. In contrast, high-power treatments (6 kW and 8 kW) caused severe disruption of mineral aggregates and substantial thermal decomposition of SOM, which led to SOM depletion, reduced soil pH and elevated EC during subsequent incubation.

Microwaves exerted disparate effects on soil bacteria and fungi. Microwave irradiation increased bacterial diversity and abundance yet reduced fungal abundance. High-power (8 kW) microwave enriched thermotolerant bacilli but inhibited actinomycetes and mycorrhizal fungi, whereas the 4 kW treatment maintained high abundances of Rhizophagus and Mucoromycota. RDA results confirmed that neutral pH and sufficient SOM supported the colonization of some beneficial microorganisms, while low pH and high EC facilitated the propagation of certain pathogenic fungi. Differential gene analysis revealed that the 4 kW treatment upregulated functional genes related to nutrient mineralization and antifungal metabolism; 6 kW mainly activated nutrient transformation genes, while 8 kW triggered microbial thermal damage repair pathways. Excessive SOM consumption and pH decline under 8 kW microwave treatment disrupted the stability of soil microecology.

In summary, 4 kW represents the optimal microwave parameter for mitigating sugarcane continuous cropping obstacles, which balances soil physicochemical conditions, activates latent nutrients and stabilizes beneficial microbial communities. From an application perspective, 915 MHz industrial microwave can serve as a physical remediation approach for green soil remediation and sustainable agriculture. As an environmentally friendly physical remediation technology without exogenous chemical inputs, it avoids secondary pollution induced by chemical agents and meets the requirements of clean agricultural production. This study improves the theoretical framework of microwave-based soil remediation and provides critical technical parameters for in situ field soil remediation. Nevertheless, the findings of this study are obtained from laboratory incubation simulations. Further field verification trials are required to test the practical performance of these microwave parameters under field conditions and clarify the interactions between microwave-induced changes in soil properties and sugarcane growth.

## Figures and Tables

**Table 1:** Thermal characteristic parameters of soil under different microwave power treatments.

Group	T_5_%/°C	T_max_/°C	W_800°C_/%
CK	342.6 ± 3.88 ^a^	481.3 ± 2.78 ^a^	94.2 ± 0.12 ^a^
2 kW	318.5 ± 4.29 ^b^	457.8 ± 7.23 ^b^	92.7 ± 0.16 ^b^
4 kW	305.2 ± 4.12 ^c^	441.6 ± 4.53 ^c^	92.1 ± 0.16 ^c^
6 kW	293.7 ± 2.90 ^d^	428.4 ± 2.53 ^d^	91.9 ± 0.12 ^d^
8 kW	325.9 ± 2.49 ^b^	463.2 ± 2.65 ^b^	97.5 ± 0.08 ^b^

Note: Different lowercase letters within the same column indicate significant differences between treatments (*p* < 0.05). T_5_% refers to the temperature when the sample mass loss reaches 5% of its initial weight, representing the initial thermal decomposition temperature and reflecting the early thermal stability of the sample. T_max_ stands for the temperature corresponding to the maximum mass loss rate (peak temperature of the DTG curve), which characterizes the thermal stability during the main decomposition stage. W_800°C_ denotes the residual mass percentage of the sample when heated to 800 °C, corresponding to the retention amount of high-temperature inorganic mineral residues.

**Table 2:** The effect of different microwave treatments on soil physicochemical properties.

Group	TN (g/kg)	TP (g/kg)	TK (g/kg)	AN (mg/kg)	AP (mg/kg)	AK (mg/kg)	SOM (g/kg)	EC (μS/cm)	pH
CK0	1.23 ± 0.02 ^a^	0.70 ± 0.03 ^a^	18.77 ± 0.29 ^a^	89.26 ± 1.70 ^bc^	12.33 ± 0.58 ^g^	125.26 ± 2.48 ^h^	22.26 ± 0.28 ^a^	0.92 ± 0.01 ^bcd^	7.22 ± 0.02 ^a^
CK15	1.21 ± 0.02 ^ab^	0.69 ± 0.04 ^ab^	18.53 ± 0.18 ^ab^	89.82 ± 0.21 ^b^	12.35 ± 0.29 ^g^	125.79 ± 1.57 ^h^	21.99 ± 0.35 ^a^	0.94 ± 0.02 ^abc^	7.21 ± 0.01 ^a^
CK30	1.20 ± 0.01 ^ab^	0.68 ± 0.04 ^ab^	18.35 ± 0.15 ^ab^	88.93 ± 0.19 ^bc^	12.23 ± 0.17 ^g^	124.54 ± 0.76 ^h^	21.76 ± 0.16 ^a^	0.95 ± 0.01 ^ab^	7.20 ± 0.01 ^a^
2 kW0	1.18 ± 0.07 ^abc^	0.63 ± 0.02 ^abc^	18.19 ± 0.25 ^bc^	85.47 ± 1.44 ^d^	13.25 ± 0.36 ^f^	133.42 ± 2.06 ^g^	21.89 ± 0.43 ^a^	0.81 ± 0.01 ^fg^	7.18 ± 0.02 ^a^
2 kW15	1.14 ± 0.03 ^bcd^	0.61 ± 0.01 ^bcd^	17.60 ± 0.08 ^de^	92.03 ± 0.22 ^a^	14.24 ± 0.24 ^de^	143.45 ± 1.17 ^e^	21.16 ± 0.53 ^b^	0.79 ± 0.01 ^g^	7.17 ± 0.02 ^a^
2 kW30	1.11 ± 0.05 ^cde^	0.60 ± 0.04 ^cde^	17.24 ± 0.14 ^ef^	89.47 ± 0.29 ^bc^	13.85 ± 0.22 ^e^	139.46 ± 0.88 ^f^	20.72 ± 0.11 ^b^	0.80 ± 0.02 ^g^	7.16 ± 0.02 ^a^
4 kW0	1.13 ± 0.02 ^bcd^	0.58 ± 0.02 ^cde^	17.90 ± 0.12 ^cd^	78.67 ± 1.50 ^e^	14.30 ± 0.25 ^de^	139.76 ± 1.62 ^f^	19.68 ± 0.26 ^c^	0.74 ± 0.01 ^h^	7.05 ± 0.04 ^b^
4 kW15	1.07 ± 0.03 ^de^	0.55 ± 0.07 ^def^	16.99 ± 0.26 ^f^	87.82 ± 0.38 ^c^	15.96 ± 0.14 ^ab^	155.44 ± 1.54 ^a^	18.65 ± 0.18 ^d^	0.72 ± 0.01 ^hi^	7.03 ± 0.04 ^b^
4 kW30	1.04 ± 0.03 ^ef^	0.53 ± 0.01 ^efg^	16.45 ± 0.22 ^g^	84.68 ± 0.29 ^d^	15.39 ± 0.16 ^bc^	149.89 ± 0.93 ^bc^	18.06 ± 0.20 ^de^	0.71 ± 0.02 ^hi^	7.02 ± 0.04 ^b^
6 kW0	1.04 ± 0.03 ^ef^	0.52 ± 0.01 ^efg^	17.40 ± 0.16 ^ef^	71.22 ± 1.37 ^g^	15.41 ± 0.27 ^bc^	146.20 ± 1.66 ^cde^	18.16 ± 0.41 ^de^	0.79 ± 0.01 ^g^	6.92 ± 0.07 ^cd^
6 kW15	0.95 ± 0.03 ^g^	0.48 ± 0.04 ^fgh^	15.98 ± 0.26 ^h^	74.52 ± 0.24 ^f^	16.13 ± 0.23 ^a^	152.86 ± 2.18 ^ab^	16.64 ± 0.15 ^f^	0.83 ± 0.02 ^ef^	6.89 ± 0.06 ^d^
6 kW30	0.91 ± 0.03 ^gh^	0.46 ± 0.01 ^gh^	15.29 ± 0.17 ^i^	67.43 ± 0.11 ^i^	14.60 ± 0.16 ^d^	138.30 ± 2.20 ^f^	15.92 ± 0.07 ^g^	0.89 ± 0.02 ^d^	6.91 ± 0.07 ^cd^
8 kW0	0.98 ± 0.02 ^fg^	0.49 ± 0.05 ^fgh^	17.20 ± 0.21 ^ef^	69.39 ± 0.63 ^h^	15.65 ± 0.16 ^abc^	147.34 ± 2.11 ^cd^	17.88 ± 0.30 ^e^	0.85 ± 0.01 ^e^	7.01 ± 0.03 ^b^
8 kW15	0.86 ± 0.05 ^hi^	0.42 ± 0.02 ^hi^	15.11 ± 0.08 ^i^	67.89 ± 0.15 ^hi^	15.31 ± 0.14 ^c^	143.91 ± 2.09 ^de^	15.69 ± 0.13 ^g^	0.91 ± 0.02 ^cd^	6.99 ± 0.03 ^bc^
8 kW30	0.80 ± 0.04 ^i^	0.39 ± 0.04 ^i^	14.08 ± 0.22 ^j^	62.35 ± 0.12 ^j^	14.06 ± 0.17 ^de^	132.16 ± 1.42 ^g^	14.62 ± 0.15 ^h^	0.97 ± 0.01 ^a^	6.98 ± 0.03 ^bc^

Note: Different lowercase letters within the same column indicate significant differences between treatments (*p* < 0.05). TN: total nitrogen (g/kg), TP: total phosphorus (g/kg), TK: total potassium (g/kg), AN: available nitrogen (mg/kg), AP: available phosphorus (mg/kg), AK: available potassium (mg/kg), SOM: soil organic matter (g/kg), EC: electrical conductivity (μS/cm), pH: soil pH. Specifically, CK0, 2 kW0, 4 kW0, 6 kW0 and 8 kW0 stood for soil samples collected at 0 day of incubation immediately after microwave treatment; CK15, 2 kW15, 4 kW15, 6 kW15 and 8 kW15 represented samples gathered after 15 weeks of incubation; CK30, 2 kW30, 4 kW30, 6 kW30 and 8 kW30 denoted samples harvested at 30 weeks of incubation.

**Table 3:** Soil bacterial and fungal microbial alpha diversity index.

Group	Bacteria				Fungi			
	Chao1	Simpson	Shannon	Pielou	Chao1	Simpson	Shannon	Pielou
CK0	1512.35 ± 25.79 ^bcde^	0.86 ± 0.00 ^bcd^	5.66 ± 0.03 ^bc^	0.54 ± 0.00 ^abc^	116.18 ± 11.88 ^bc^	0.81 ± 0.02 ^abc^	3.55 ± 0.00 ^ab^	0.55 ± 0.01 ^ab^
CK15	1377.81 ± 18.75 ^dfg^	0.82 ± 0.02 ^d^	4.97 ± 0.07 ^c^	0.48 ± 0.01 ^c^	105.07 ± 3.73 ^bc^	0.50 ± 0.05 ^d^	1.84 ± 0.16 ^d^	0.30 ± 0.03 ^e^
CK30	1217.60 ± 9.32 ^h^	0.81 ± 0.00 ^d^	4.96 ± 0.06 ^c^	0.49 ± 0.00 ^c^	81.78 ± 0.22 ^bc^	0.58 ± 0.09 ^d^	2.19 ± 0.36 ^cd^	0.35 ± 0.06 ^de^
2 kW0	1595.12 ± 22.27 ^bc^	0.86 ± 0.00 ^bcd^	5.80 ± 0.08 ^abc^	0.55 ± 0.01 ^abc^	127.76 ± 5.01 ^a^	0.85 ± 0.00 ^a^	4.02 ± 0.19 ^a^	0.62 ± 0.02 ^a^
2 kW15	1412.66 ± 98.78 ^defg^	0.81 ± 0.02 ^d^	5.05 ± 0.16 ^c^	0.49 ± 0.01 ^c^	106.14 ± 8.74 ^bc^	0.68 ± 0.03 ^abcd^	2.62 ± 0.05 ^cd^	0.41 ± 0.00 ^de^
2 kW30	1404.06 ± 79.99 ^defg^	0.82 ± 0.02 ^cd^	5.10 ± 0.33 ^c^	0.49 ± 0.03 ^c^	128.94 ± 39.56 ^a^	0.62 ± 0.06 ^bcd^	2.41 ± 0.23 ^cd^	0.37 ± 0.01 ^de^
4 kW0	1666.23 ± 77.55 ^ab^	0.94 ± 0.00 ^a^	6.14 ± 0.11 ^ab^	0.58 ± 0.01 ^ab^	93.21 ± 1.12 ^bc^	0.81 ± 0.02 ^abc^	3.66 ± 0.08 ^a^	0.59 ± 0.01 ^ab^
4 kW15	1542.44 ± 57.56 ^bcd^	0.88 ± 0.01 ^abc^	5.69 ± 0.20 ^bc^	0.54 ± 0.02 ^abc^	83.31 ± 5.31 ^bc^	0.67 ± 0.01 ^abcd^	2.39 ± 0.01 ^cd^	0.40 ± 0.01 ^de^
4 kW30	1484.06 ± 44.94 ^cdef^	0.86 ± 0.02 ^bcd^	5.29 ± 0.07 ^bc^	0.51 ± 0.01 ^bc^	113.66 ± 23.54 ^bc^	0.69 ± 0.06 ^abcd^	2.71 ± 0.22 ^cd^	0.42 ± 0.05 ^cde^
6 kW0	1766.30 ± 1.49 ^a^	0.91 ± 0.04 ^ab^	5.55 ± 0.78 ^bc^	0.52 ± 0.07 ^bc^	91.70 ± 4.40 ^bc^	0.83 ± 0.02 ^ab^	3.73 ± 0.12 ^a^	0.62 ± 0.03 ^a^
6 kW15	1505.99 ± 21.55 ^cde^	0.92 ± 0.02 ^ab^	5.75 ± 0.10 ^bc^	0.55 ± 0.01 ^abc^	69.50 ± 6.50 ^c^	0.63 ± 0.10 ^bcd^	2.40 ± 0.27 ^cd^	0.42 ± 0.04 ^cde^
6 KW30	1503.44 ± 41.63 ^cde^	0.88 ± 0.02 ^abcd^	5.68 ± 0.16 ^bc^	0.54 ± 0.01 ^abc^	98.03 ± 8.75 ^bc^	0.68 ± 0.05 ^abcd^	2.65 ± 0.24 ^cd^	0.42 ± 0.05 ^cde^
8 kW0	1764.49 ± 10.56 ^a^	0.94 ± 0.01 ^a^	6.57 ± 0.15 ^a^	0.61 ± 0.01 ^a^	117.08 ± 3.83 ^bc^	0.79 ± 0.07 ^abc^	3.55 ± 0.43 ^ab^	0.54 ± 0.05 ^abc^
8 kW15	1330.89 ± 21.86 ^fgh^	0.83 ± 0.01 ^cd^	5.19 ± 0.11 ^c^	0.50 ± 0.01 ^c^	86.45 ± 11.02 ^bc^	0.65 ± 0.11 ^bcd^	2.80 ± 0.44 ^bc^	0.47 ± 0.05 ^bcd^
8 kW30	1300.39 ± 52.62 ^gh^	0.82 ± 0.03 ^cd^	5.07 ± 0.23 ^c^	0.49 ± 0.02 ^c^	116.03 ± 30.94 ^bc^	0.61 ± 0.05 ^cd^	2.26 ± 0.43 ^cd^	0.35 ± 0.06 ^de^

Note: Different lowercase letters within the same column indicate significant differences between treatments (*p* < 0.05). Specifically, CK0, 2 kW0, 4 kW0, 6 kW0 and 8 kW0 stood for soil samples collected at 0 days of incubation immediately after microwave treatment; CK15, 2 kW15, 4 kW15, 6 kW15 and 8 kW15 represented samples gathered after 15 weeks of incubation; CK30, 2 kW30, 4 kW30, 6 kW30 and 8 kW30 denoted samples harvested at 30 weeks of incubation.

## Data Availability

The data presented in this study are openly available in zenodo at 10.5281/zenodo.21466149.

## Supplementary Materials

The following supporting information can be downloaded at https://zenodo.org/records/21466150 (Video S1 was accessed on 15 July 2026); Video S1: The temperature rise process of soil under different microwave powers for 0–10 min.
